# Inhibiting Bacterial Drug Efflux Pumps via Phyto-Therapeutics to Combat Threatening Antimicrobial Resistance

**DOI:** 10.3389/fmicb.2018.02990

**Published:** 2018-12-10

**Authors:** Varsha Shriram, Tushar Khare, Rohit Bhagwat, Ravi Shukla, Vinay Kumar

**Affiliations:** ^1^Department of Botany, Prof. Ramkrishna More College, Savitribai Phule Pune University, Pune, India; ^2^Department of Biotechnology, Modern College of Arts, Science and Commerce (Savitribai Phule Pune University), Pune, India; ^3^Department of Environmental Science, Savitribai Phule Pune University, Pune, India; ^4^Centre for Advanced Materials and Industrial Chemistry, School of Science, RMIT University, Melbourne, VIC, Australia

**Keywords:** antimicrobial resistance, efflux pumps, efflux pump inhibitors, phyto-therapeutics, drug resistance reversal

## Abstract

Antibiotics, once considered the lifeline for treating bacterial infections, are under threat due to the emergence of threatening antimicrobial resistance (AMR). These drug-resistant microbes (or superbugs) are non-responsive to most of the commonly used antibiotics leaving us with few treatment options and escalating mortality-rates and treatment costs. The problem is further aggravated by the drying-pipeline of new and potent antibiotics effective particularly against the drug-resistant strains. Multidrug efflux pumps (EPs) are established as principal determinants of AMR, extruding multiple antibiotics out of the cell, mostly in non-specific manner and have therefore emerged as potent drug-targets for combating AMR. Plants being the reservoir of bioactive compounds can serve as a source of potent EP inhibitors (EPIs). The phyto-therapeutics with noteworthy drug-resistance-reversal or re-sensitizing activities may prove significant for reviving the otherwise fading antibiotics arsenal and making this combination-therapy effective. Contemporary attempts to potentiate the antibiotics with plant extracts and pure phytomolecules have gained momentum though with relatively less success against Gram-negative bacteria. Plant-based EPIs hold promise as potent drug-leads to combat the EPI-mediated AMR. This review presents an account of major bacterial multidrug EPs, their roles in imparting AMR, effective strategies for inhibiting drug EPs with phytomolecules, and current account of research on developing novel and potent plant-based EPIs for reversing their AMR characteristics. Recent developments including emergence of *in silico* tools, major success stories, challenges and future prospects are also discussed.

## Introduction

Antimicrobial resistance (AMR) or ineffectiveness of commonly used drugs/antibiotics against specific bacteria has emerged as one of the most threatening human health concerns and a major challenge for global drug discovery programs. AMR (also known as drug resistance) has been reported at three increasing levels, multidrug resistance (MDR), extensive drug resistance (XDR) and pan-drug resistance (PDR). By definition, MDR stands for acquisition of non-susceptibility to at least one agent in three or more antimicrobial classes, while XDR shows the non-susceptibility to at least one agent in all, except two or fewer antimicrobial classes, while PDR implies non-susceptibility to all antimicrobial agents from all available classes (Exner et al., 2017; Spengler et al., 2017). AMR is threatening millions of lives worldwide, and is rightly declared as a *global risk* by the World Economic Forum (World Economic Forum, 2013). Since the very first report on AMR in *Enterobacteria* in 1950s (Watanabe, 1963; Levy, 2001), many drug-resistant strains have been reported and their number as well as the resistance level is on the rise. Though several classes of antibiotics were discovered in the antibiotic era (Table 1), we are heading to a post-antibiotic era, where an increasing number of previously curable infections are turning into non-curable and life-threatening (Spengler et al., 2017). Though development of AMR or antibiotic resistance is a natural phenomenon, irrational use of antibiotics speed-ups the emergence of drug-resistant strains (World Health Organization, 2014). Once the AMR is gained by the bacteria, it is successively transmitted to the next progeny via vertical gene transfer or other bacteria through horizontal gene transfer process, making their treatment more difficult (Chandra et al., 2017).

**Table 1:** Classes of commonly used antibiotics along with their examples and corresponding modes of action.

**Class of antibiotics**				**Examples**	**Mode of action**
Beta Lactams	Beta lactamase inhibitors			Sulbactam, Tazobactam, Clavulanic acid, Avibactam	Cell wall synthesis inhibitors
	Penicillins	Penicillinase sensible	Aminopenicillins	Ampicillin, Amoxicillin	
			Natural penicillins	Penicillin G, Penicillin VK	
		Penicillinase resistant		Nafcillin, Dicloxacillin, Oxacillin	
		Anti-pseudomonal	Carboxypenicillins	Ticarcillin, Carbenicillin	
			Ureidopenicillins	Piperacillin, Mezlocillin, Azlocillin	
	Cephalo-sporins	1st generation		Cephalexine, Cefadroxil, Cefazolin, Cephradrine	
		2nd generation		Cefuroxime, Cefoxitin, Cefaclor, Loracarbef, Cefprozil	
		3rd generation		Cefoperazone, Cefpodoxime, Ceftriaxone, Cefotaxime, Ceftazidime	
		4th generation		Cefepime, Cefpirome	
		5th generation		Ceftaroline, Ceftolozane	
	Carbapenems			Meropenem, Doripenem, Ertapenem, Imipenem	
	Monobactams			Aztreonam	
No lactams	Glycopeptides			Vancomycin, Dalbavancin, Telavancin, Oritavancin	
	Other			Colistin, Daptomycin, Polymixin B, Isoniazid	
	Amino-glycosides			Amikacin, Streptomycin, Neomycin, Gentamicin, Tobramycin	Protein synthesis inhibitors
	Tetracyclins			Doxycyclin, Tetracyclin, Democlocyclin, Minocyclin, Tigecyclin	
	Oxazolidonones			Linezolid, Tidezolid	
	Streptogramins			Quinupristin	
				Chloramphenicol	
	Macrolides			Erythromycin, Clarithromycin, Azithromycin	
	Lincosamides			Clindamycin, Lincomycin	
	Fluorquinolones			Ciprofloxacin, Sparfloxacin, Levofloxacin, , Norfloxacin	DNA
	Quinolones			Nalidixic acid	topoisomerases inhibitors
	Sulfonamides			Sulfamethoxazole, Sulfasalazine, Ag sulfadiazine, Sulfisoxazole	Folic acid synthesis inhibitor
	DHFR inhibitors			Trimethoprim, Pyrimethamine	
	Nitroimidazoles			Metronidazole, Tinidazole	DNA Damage
				Rifampin	mRNA synthesis

The drug resistance characteristics may be attributed to the abilities of such strains in fast altering their genetic make-up or inducing epigenetic changes (Davies and Davies, 2010; Motta et al., 2015; Rahman et al., 2017). Necessary adaptations are achieved by bacteria to-respond-to and to counteract the antibiotics either via procurement of foreign genetic material encoding resistance via horizontal gene transfer or mutations in drug-targets / antibiotics-degrading enzymes and alterations in permeability of the outer bacterial membrane. There is an unprecedented upsurge in bacterial strains with elevated AMR in both Gram-negative and Gram-positive phenotypes. The ESKAPE (*Enterococcus faecium, Staphylococcus aureus, Klebsiella pneumoniae, Acinetobacter baumannii, Pseudomonas aeruginosa*, and *Enterobacter* species) pathogens have emerged with high degree of AMR and are major cause of life-threatening nosocomial infections (Santajit and Indrawattana, 2016). Even other strains like *Escherichia coli, Shigella* species, *Neisseria gonorrhoeae*, and *Proteus mirabilis* have shown significant levels of AMR (Fair and Tor, 2014; Prasch and Bucar, 2015; Cerceo et al., 2016). Development of resistance against the carbapenem, a class of highly effective antibiotics and regarded as the last line of defense against pathogenic Gram-negative bacteria hints at the alarming situation (Kumarasamy et al., 2010; Dwivedi et al., 2015).

**Figure 1:**
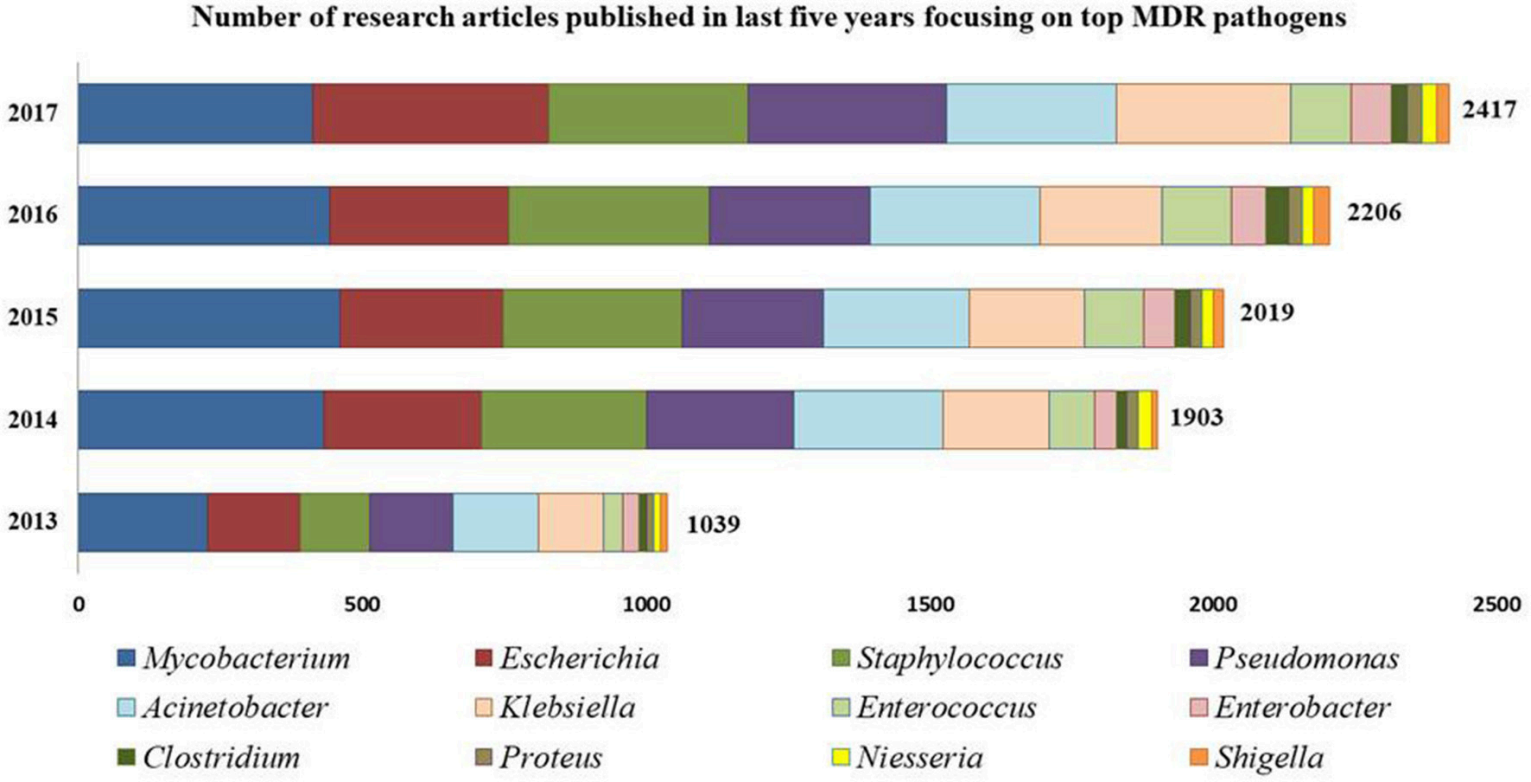
Number of research articles reported from 2013 to 2017 focusing on MDR bacterial strains. [Source: PubMed; Key words used: Multidrug resistant “*Genus name*”].

Intrinsically, AMR is more prevalent and severe in Gram-negative bacteria than their Gram-positive counterparts due mainly to the outer membranes serving as permeability barrier for drug-influx into the Gram-negative bacteria (Silhavy et al., 2010; Exner et al., 2017). To attain low sensitivity against biocidal compounds, Gram-negative bacteria reduce their outer membrane permeability by reducing the number of porins and inducing drug efflux pumps (EPs) for outward transport of drug molecules, often in a non-specific manner making the bacterial cells resistant to multiple antibiotics (Masi et al., 2017). However, despite these morphological differences, Gram-positive bacteria cannot be ignored or underestimated and noteworthy examples include methicillin resistant *S. aureus* (MRSA) and vancomycin resistant *S. aureus* (VRSA), coagulase negative *Staphylococci* members including *S. epidermidis* and *S. haemolyticus, Streptococcus pneumonia, E. faecalis, E. faecium*, and *Clostridium difficile* (Schindler and Kaatz, 2016). Figure 1 shows a gradual upsurge in the number of research articles focused on most-prevalent MDR strains.

**Figure 2:**
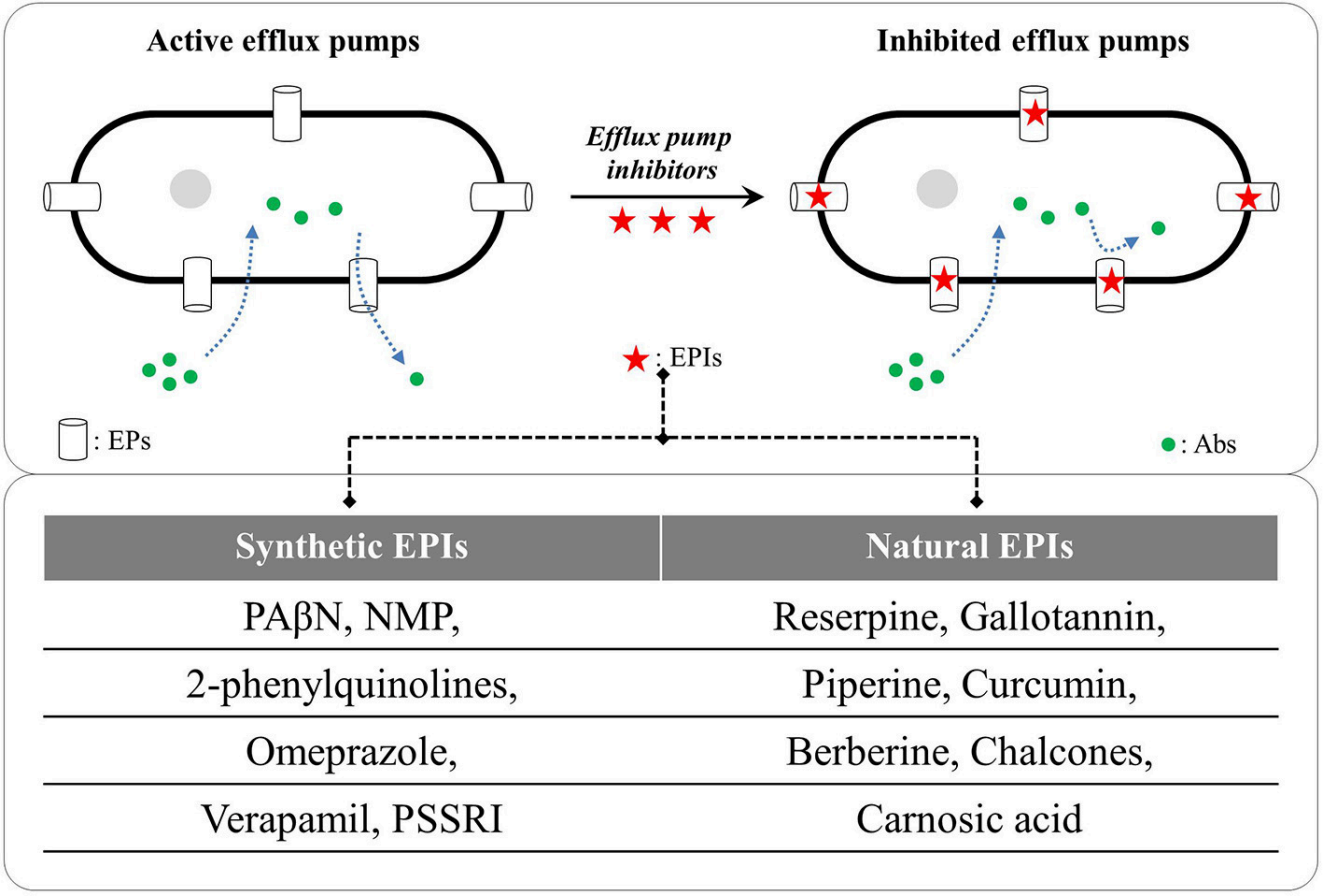
Examples of synthetic and natural efflux pump inhibitors (EPIs); PAβN: phenyl-arginine-β-naphthylamide, NMP: 1-(1- naphthylmethyl)-piperazine, PSSRIs: phenylpiperidine selective serotonin re-uptake inhibitors (German et al., 2008; Hannula and Hänninen, 2008; Li et al., 2015; Ni et al., 2016; Willers et al., 2016; Sabatini et al., 2017). Abs, antibiotics; EPs, efflux pumps.

In recent years, EPs have emerged as key drivers for AMR in Gram-negative and Gram-positive bacteria, and therefore, are looked upon as potent and universal targets for containing the drug-resistant phenotypes. EPs are vital in other physiological processes also including stress-adaptations, virulence, pathogenicity and transportation of essential nutrients (Piddock, 2006; Fernandez and Hancock, 2012; Costa et al., 2013; Kourtesi et al., 2013; Sun et al., 2014). Identifying novel and potent EP inhibitors (EPIs) to revert the AMR is therefore gaining momentum. EPIs are the compounds with capability to reduce resistance or a complete reversal of AMR against otherwise ineffective antibiotics via inhibiting the EPs (Sun et al., 2014; Gill et al., 2015; Wright, 2016; Spengler et al., 2017). The first EPI against RND-type EPs was reported by Lomovskaya et al. (2001) a phenylalanine-arginine β-naphthylamide (PAβN) effective against Mex pumps in *P. aeruginosa* and AcrAB-TolC pump in *E. coli*. Since then, various synthetic and natural compounds have been screened for their EPI capabilities (reviewed by Prasch and Bucar, 2015; Spengler et al., 2017; Shin et al., 2018; Yang et al., 2018). Figure 2 illustrates the inhibition of microbial drug efflux via synthetic and natural EPIs.

Medicinal plants with antimicrobial properties have the potential to serve as the reservoir of novel and effective EPIs (Newman and Cragg, 2012). Though there are several reports on assessing medicinal plants for their antimicrobial properties (crude extracts and occasionally pure molecules), few investigations were aimed against MDR/XDR strains and fewer on deciphering the underlying resistance mechanisms targeted by the plant products (Kumar et al., 2013). But newer studies are coming up for identifying novel phytomolecules capable of reversing EP-mediated AMR. Some striking phytochemicals which have recently been identified for their EPI potentials include catechol, pinene, gingerol, capsaicin, resveratrol and the number is increasing (Prasch and Bucar, 2015).

In this review, we are presenting an account of major EPs, their roles in imparting bacterial AMR, strategies for identifying plant-based EPIs emphasizing on the potent phyto-EPIs active at relatively lower concentrations, reported during the last 8 years. High throughput screening and *in silico* approaches for predicting the EPIs and their binding targets/sites are also discussed.

## Physiological Roles Played by Bacterial Efflux Pumps

Bacterial genome comprises of EP genes, expressed under tight regulation of global/local transcription factors (e.g., BmrR: transcriptional regulator of efflux pump Bmr in *B. subtilis*; QacR: transcriptional repressor of QacA transporter in *S. aureus;* AcrR: transcription repressor of acrB efflux pump in *E. coli*) proposing the important physiological roles the EPs play during cell development, stress adaptations and bacterial pathogenesis (Sun et al., 2014). The knowledge about these regulatory mechanisms may advance the understandings of physiologically originated AMR, frequently observed in nature (Sun et al., 2014). As discussed above, bacterial EPs have a tremendous capacity to extrude the variety of toxic compounds, needed for the cell survival in a given physiological niche and are vital for maintaining pathogenicity. This is further supported by the studies showing reduced pathogenicity in the bacterial strains lacking EPs. Buckley et al. (2006) showed that *S. typhimurium acrB* or *tolC* deficient mutant poorly colonized in the avian gut, highlighting the requirement of complete AcrAB-TolC system for virulence. The *S. typhimurium* strain lacking all the drug efflux assemblies became avirulent, when tested in a mouse model (Nishino et al., 2006). To confirm the role of EPs in bacterial pathogenesis, Hirakata et al. (2002) assessed the ability of EP (MexAB-OprM, MexCD-OprJ, MexEF-OprM, and MexXY-OprM) mutants of *P. aeruginosa* to invade Madin-Darby canine kidney cells. The findings revealed that except mexCD-OprJ, all other systems evidenced decreased bacterial invasion abilities.

EPs are also known to effect the bacterial cell communication during the stress-responses, especially in the quorum-sensing (González and Keshavan, 2006). As transportation of auto-inducers (chemical signals generated during quorum sensing) is a key-event during cell-cell interactions via quorum-sensing, drug EPs assist their transport across the membrane (Liang et al., 2016). Moore et al. (2014) confirmed a vital role played by MexAB-OprM efflux system in the secretion of a major auto-inducer N-acylated L-homoserine lactone by *P. aeruginosa* cells. The study also postulated this auto-inducer as a substrate for MexAB-OprM system (Moore et al., 2014). Further, Martinez et al. (2009) advocated the EP-mediated ceasing of quorum-sensing via augmented efflux of auto-inducers, facilitating quick bacterial response to stress signals. One more physiological role attributed to EPs is in the biofilm formation. Recent studies confirm the involvement of many well-characterized efflux systems, AcrAB-TolC of *E. coli*, AcrD of *S. enterica*, AdeFGH of *A. baumannii* and MexAB-OpeM of *P. aeruginosa* in biofilm formation (Alav et al., 2018). Kvist et al. (2008) reported an up-regulation of 20 genes encoding EP-transporters in *E. coli* during the growth of biofilm. Similarly, the EP-mediated export of colanic acid for capsule-matrix formation was observed along with up-regulated TetA(C) in *E. coli*, facilitating the biofilm formation (May et al., 2009). Collectively, the physiological roles of EPs are vital for pathogenic stability and virulence maintenance in bacteria.

The synthetic EPIs namely carbonylcyanide m-chlorophenylhydrazone (CCCP), chlorpromazine and PAβN were reported to prevent biofilm formation in *E. coli, P. aeruginosa* and *S. aureus* (Baugh et al., 2014). However, investigations on evaluating phytochemicals for their anti-biofilm potencies via inhibiting EPs are few. Fiamegos et al. (2011) isolated 4,5-O-dicaffeoylquinic acid from *Artemisia absinthium* which was proved to be a potent inhibitor of MFS pumps in *E. coli* and *E. faecalis* and as an anti-biofilm agent (Fiamegos et al., 2011). Recent reports advocate that nanomaterials in combination with phyto-EPIs can also be an effective therapy for containing drug-resistant infections (Gupta et al., 2017).

## Bacterial Efflux Pumps: The Warheads in AMR Strains

Though bacterial AMR has several origins and many adaptive mechanisms are employed by drug-resistant strains against the antibiotics, the intrinsic EPs hold the key. Recent clinical and laboratory data establish that bacterial EPs are not only critical for drug-extrusion but also contribute to their virulence and adaptive responses (Du et al., 2018). Often, antimicrobial drug exposure induces intricate bacterial reactions including altered expressions of several genes encoding the transporters, as revealed by the phenotypic profiling of *E. coli* (Nichols et al., 2011).

Bacterial EPs are acknowledged either as primary active transporters using ATPs as an energy source, or as secondary active transporters acquired due to electrochemical potential difference created by pumping out Na^+^ and H^+^ outside the membrane (Dwivedi et al., 2017a).

This may be considered for classifying EPs into two broad super-families namely; ATP-binding cassette (ABC) multidrug transporters and secondary transporters using proton motive force (PMF) as an energy source (Putman et al., 2000). The second super-family can again be categorized in four subclasses, the major facilitator superfamily (MFS), resistance-nodulation-cell division (RND), multidrug and toxic compound extrusion (MATE) and small-MDR (SMR) family (Fernandez and Hancock, 2012; Sun et al., 2014). RND and MFS pumps are the most common in bacteria. With relatively narrow spectrum of specificity, MFS pumps are found in both Gram-negative and Gram-positive bacteria; while poly-selective RND pumps are exclusive to Gram-negative bacteria (Ward et al., 2001; Molnár et al., 2010). The SMR transporters show specificity for broad-spectrum polyaromatic cations convening resistance for compounds sharing similar chemical description. The MATE transporters are similar in size to MFS transporters but they do not share any sequence similarity with them (Jack et al., 2001). Apart from the classified super-families and sub-families, the MATE, SMR, and RND classes are distributed uniquely to prokaryotes whereas MFS and ABC transporters are dispersed in both prokaryotes and eukaryotes.

The large MFS is one of the most functionally diverse transporter families comprising multiple transportation types for drugs as well as sugars. These transporters comprise ~400 amino acids arranged as membrane-spanning helices (Saier et al., 1998). Based on the helical structure, MFS transporters can be classified as either 12-[e.g., TatA(B): class B tetracyclin transporter from *E. coli*] or 14-helix transporters [e.g., TatA(K): class K tetracyclin transporter from *S. aureus*], and TetA(B) is one of the most extensively studied members of the family (Lynch, 2006).

The SMR pumps represent smallest multidrug transporters, possessing only four trans-membrane helices without any extra membrane domain. But single minimal functional SMR unit represents eight helices as these are functionally active in dimeric form (Higgins, 2007). Well-illustrated example from this family is the electromagnetic antiporter EmrE from *E. coli*, responsible for resistance to a range of cationic-hydrophobic entities including antibiotics.

The latest structurally characterized class of EPs is MATE, involved in various vital biological functions (He et al., 2010). These transporters are equivalent to MFS transporters with a typical composition of ~450 amino acids putatively arranged in 12 helices; but with no sequence similarity with MFS counterparts (Jack et al., 2001). Some characterized MATE members include NorM from *N. gonorrhoeae* and *N. meningitides* and YdhE from *E. coli*. However, limited structural and functional knowledge is available about this family (Dwivedi et al., 2017a).

Though EPs from other families contribute to the AMR against certain antibiotics, RND pumps are the most potent drug efflux systems conferring resistance against clinically important antibiotics and biocides. Members of this family are known for their roles against a wide range of molecules with dissimilar structures including antibiotics, biocides, organic solvents, antimicrobial peptides, detergents, dyes, and bile salts (Poole, 2004). The tripartite complex of pumps from this family comprises inner membrane protein (IMP), outer membrane protein (OMP) along with a periplasmic membrane fusion protein (MFP) as a connector (Venter et al., 2015). The best understood tripartite complexes include AcrA-AcrB-TolC from *E. coli* and MexA-MexB-OprM from *P. aeruginosa* (Du et al., 2013, 2014). Greater structural and functional similarities between IMPs of these two systems are described by Welch et al. (2010). Exploration on biochemical and structural aspects of AcrB has revealed that these IMPs contain proximal and distal binding pockets, divided by G-loop (with 614–621 residues). Conformational flexibility of G-loop is crucial for movement of substrate along the binding site (Eicher et al., 2012; Cha et al., 2014). Table 2 lists various EPs belonging to the major families from prevalent pathogenic bacteria.

**Table 2:** Examples of various efflux pumps belonging to major efflux pump families from prevalent pathogenic bacterial strains.

**Pathogen**	**EP Family**	**Example**	**Substrate**	**References**
*Acinetobacter baumannii*	ABC	*MacAB-TolC*	ML	Okada et al., 2017
	MATE	*AbeM*	ACR, AG, DAU, DOR, FQ	Su et al., 2005
	MFS	*CraA*	CHL	Roca et al., 2009
	RND	*AdeABC, AdeFGH, AdeIJK*	AG, BL, FQ, ML, TET, BIO	Coyne et al., 2010, Rajamohan et al., 2010
*Campylobacter jejuni*	RND	*CmeABC, CmeDEF*	AG, BL, CHL, FQ, ML, RIF, TET, EB	Lin et al., 2002; Akiba et al., 2006
*Enterococcus faecalis*	ABC	*EfrAB*	ACR, CIP, DAU, DOR, DOX, NOR, TPP	Lee et al., 2003
*Escherichia coli*	ABC	*MacAB-TolC*	ML	Kobayashi et al., 2001
	MFS	*MdfA*	CHL, DOR, NOR, TET	Nishino et al., 2006
		*QepA/QepA2*	FQ	Cattoir et al., 2008
	RND	*AcrAB-TolC*	BL, CHL, FQ, ML, NOV, RIF, TET, TGC, R6G	Swick et al., 2011, Lennen et al., 2013
		*OqxAB*	CHL, FQ	Hansen et al., 2004
	SMR	*EmrE*	ACR, EB, QAC	Schuldiner, 2009, Beketskaia et al., 2014
*Klebsiella pneumoniae*	MATE	*KetM*	DAPI	Ogawa et al., 2015
	MFS	*KpnGH*	CAZ, CEF, STR, TET	Srinivasan et al., 2014
	RND	*OqxAB*	CHL, FQ	Hansen et al., 2004
	SMR	*KnpEF*	BAC, CEF, CHX, ERY, STR, TET, TRI	Srinivasan and Rajamohan, 2013
*Mycobacterium tuberculosis*	ABC	*Rv1218c*	BAP, BPD, PRI, PYR	Balganesh et al., 2010, Balganesh et al., 2012
	MFS	*Tap*	PAS, SPE, TET	Ramón-García et al., 2012
	SMR	*Mmr*	CAB, CLA, TPP	Balganesh et al., 2012, Rodrigues et al., 2013
*Pseudomonas aeruginosa*	RND	*MexAB-OprM, MexXY-OprM/A, MexCD-OprJ, MexEF-OprN*	AG, BL, CHL, FQ, ML, SUL, TET, TGC, TMP, BIO, EB	Poole et al., 1996, Köhler et al., 1997
*Staphylococcus aureus*	ABC	*Isa(E)*	LIN, PLE, STA	Wendlandt et al., 2015
		*Msr(A)*	ML, TEL	Vimberg et al., 2015
	MATE	*MepA*	BIO, EB, FQ, TIG	Kaatz et al., 2005
	MFS	*NorA*	FQ	Yoshida et al., 1990
		*QacA*	ACR, CHX, EB, QAC	Littlejohn et al., 1992
*Streptococcus pneumoniae*	ABC	*PatAB*	FQ	Marrer et al., 2006
	MFS	*MefE*	ML	Tait-Kamradt et al., 1997
*Vibrio* spp.	ABC	*VcaM*	CIP, DAU, DOR, NOR, TET	Huda et al., 2003
	MATE	*NorM*	AG, EB, FQ	Morita et al., 1998
	MFS	*EmrD-3*	EB, LNZ, ERY, CHL	Bruns et al., 2017

*ACR, acriflavine; AG, aminoglycosides; BAC, benzalkonium chloride; BAP, biaryl-piperazines; BL, β-lactams; BIO, biocides; BPD, bisanilino-pyridines; CAB, cetyl-trimethyl-ammoniumbromide; CAP, cationic antibacterial peptides; CAZ, ceftazidime; CEF:cefepime; CHL, chloramphenicol; CHX, chlorhexidine; CIP, ciprofloxacin; CLA, clofazimine; DAU, daunomycin; DAPI, 4',6-diamidino-2-phenyl indole; DOR, doxorubicin; DOX, doxycycline; EB, ethidium bromide; ERY, erythromycin; FQ, fluoroquinolones; FUA, fusidic acid; LIN:lincosamide; LNZ, linezolid; ML, macrolides; NOR, norfloxacin; NOV, novobiocin; PAS, p-aminosalicylate; PLE, pleuromutilin; PRI, pridones; PYR, pyrroles; QAC, quaternary ammonium compounds; SPE, spectinomycin; STA, streptogramin A; STR, streptomycin; SUL, sulfonamides; TEL, telithromycin; TET, tetracycline; TGC, tigecycline; TMP, trimethoprim; TPP, tetraphenylphosphonium, TRI, triclosan*.

Collectively, the complex EP assemblies are critical for bacterial pathogenesis, virulence, biofilm formation, and adaptive-responses ultimately conferring and defining bacterial AMR (Piddock, 2006; Martinez et al., 2009; Baugh et al., 2012, 2014; Du et al., 2018). EPs are critical for bacterial AMR as they exclude most of the unwanted entities until the cell gets required time for acquiring resistance (Piddock, 2014; Venter et al., 2015).

## Bacterial Efflux Pumps: The Urgent Threats Requiring Immediate Remedy

It is well-established that EPs comprise one of the most vital systems in bacteria responsible for both innate and acquired AMR (Blair et al., 2015). There are reports of EPs from different superfamilies and the occurrence of various types of EPs from the same superfamily in a single bacterial species (Piddock, 2006). For instance, whole genome sequencing of the colistin resistant *Enterobacter cloacae* showed presence of multiple EPs (Norgan et al., 2016). Differential substrate profiles of EPs are also a characteristic feature which may diverge between or within the superfamily (Poole, 2005, 2007). Although the core motive of EPs related studies is focused on AMR, several reports however confirmed other but significant functions of bacterial EPs including quorum-sensing, biofilm formation, virulence, pathogenicity and bacterial behavior (Piddock, 2006; Yang et al., 2006; Fahmy et al., 2016).

Up-regulation of gene expression levels are one of the main drivers for chromosomally acquired AMR. This can be triggered due to the gene induction, activated transcription, or due to regulatory mutations (Grkovic et al., 2002). The coding region for an EP is usually found contiguous to the regulatory proteins controlling the expression levels of pump gene in response to substrates. For example, AdeL, an LTTR (LysR-type transcriptional regulator) family protein exists opposite to the adeFGH operon that regulates the expression of genes encoding RND efflux system in *A. baumannii* (Liu et al., 2018). The expression levels of EP-associated proteins along with porins is mutually controlled by several global regulatory elements, modifying the transcription patterns of EP-family transcripts either directly or through a cascade of regulatory events (Warner and Levy, 2010; Sun et al., 2014). Further, the expression of MexAB-OprM efflux system is governed by repressor protein mexR, encoded by a gene located upstream of the mexAB-oprM operon in *P. aeruginosa* (Suresh et al., 2018). Similarly, the acrAB operon system is regulated by regulator acrR in *E. coli*, located 140 bp up-stream of the acrAB operon (Ma et al., 1996).

Another striking bacterial character adding to the AMR nature is heteroresistance, the occurrence of differential responses to antibiotics by the bacterial cells from the same population, a phenomenon first reported in *S. aureus* (Kayser et al., 1970). Interestingly, drug resistant and sensitive bacterial cells may co-exist in a single culture (Morand and Mühlemann, 2007). The mechanism underlying heteroresistance acquirements are yet to be fully understood, however, the active EPs are strongly linked to heteroresistance (Chen et al., 2017). Designing a treatment course against such strains is difficult as there are high chances of increase in the frequency of resistant-bacterial-population and stimulation of cross-resistance to antimicrobial lysozymes of the host system (Napier et al., 2014; Telke et al., 2017). Up-regulation of OpxAB gene in *Salmonella typhimurium* (Chen et al., 2017) and AdeABC gene in *A. calcoaceticus-A. baumannii* (Ruzin et al., 2007) are attributed for mediating the tigecyclin heteroresistance. Similarly, colistin associated heteroresistance is also reported in *E. asburiae* LH74 and *E. cloacae* NH52 (Telke et al., 2017), showing its association with overexpression of *acrAB-tolC* EPs under the regulation of soxRS genes.

AMR phenotypes may result from concurrent acquisition of several AMR mechanisms simultaneously. It may include a combination of phenomena like chromosomally acquired resistance, multiple chromosomal changes with time, and/or a single mutational event activating the AMR mechanisms including the EPs (Lister et al., 2009). The over-expressions of EPs and their corresponding genes have been reported to contribute to MDR in *P. aeruginosa* (Shigemura et al., 2015). Recent studies have confirmed the role of EPs in fluoroquinolone resistant *E. coli* (Amabile-Cuevas et al., 2010; Swick et al., 2011; Yasufuku et al., 2011). Similarly, two fluoroquinolone resistant clinical isolates of *Shigella* showed overexpression of the TolC channels, part of AcrAB-TolC tripartite responsive to ciprofloxacin (Kim et al., 2008). These findings confirm that the up-regulation of EP genes contribute significantly to diminish intracellular antibiotics level, with a selectivity of the efflux transporter.

**Figure 3:**
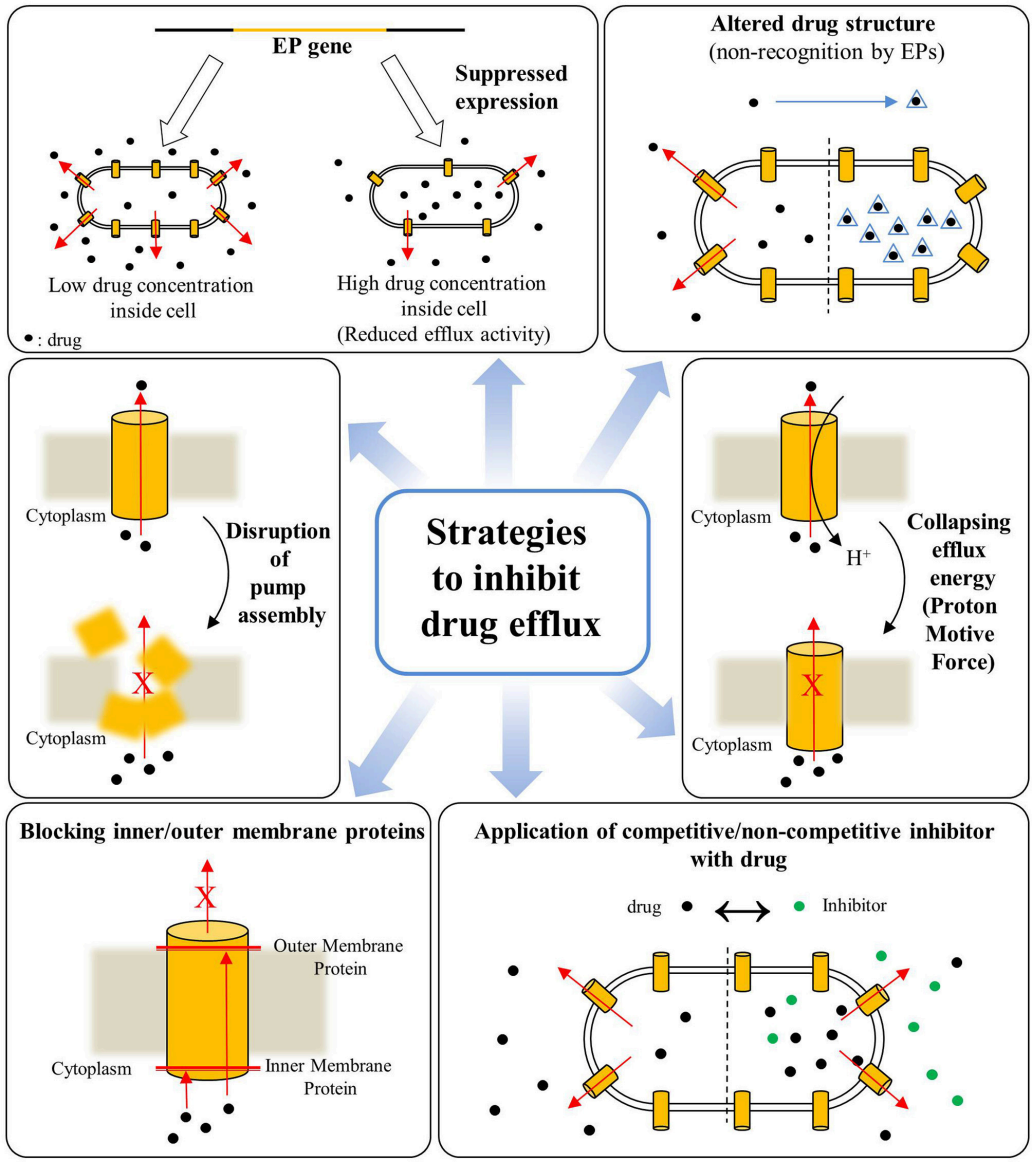
Various strategies for inhibition of drug efflux from bacterial cells for combating antimicrobial resistance (Based on reviews by Pagès and Amaral, 2009; Venter et al., 2015).

Overall, the poly-specificity of EPs, their overexpression in response to drugs along with the phenomenon of heteroresistance seem key factors responsible for drug-resistance in a wide-range of bacterial species, especially in Gram-negative bacteria making them difficult to treat with conventional drug arsenal. The drug-efflux mediated bacterial AMR is a mounting threat to global healthcare, therefore EPs are gaining unprecedented attention not only from the perspectives of basic understandings that how they work and impart drug-resistance but also as emerging targets for development of novel and potent adjunct-therapies for combating AMR in community and nosocomial infections. As a result, inhibition of drug efflux from bacterial cells via inhibiting or disrupting the EPs is an emerging approach for combating the threatening AMR. Various approaches have been developed in recent past and a schematic for these strategies for inhibition or disruption of bacterial drug efflux is illustrated in Figure 3.

## Phytotherapeutics–The Potent Efflux Pump Inhibitors

Phytochemicals are critical for human health-care since ancient times. Medicinal plants are hailed as a reservoir for phytochemicals capable of providing new and potent drug leads to contain the AMR via targeting the principal determinants of drug-resistance including EPs (Newman and Cragg, 2012; Prasch and Bucar, 2015). This review focuses mainly on EPIs of plant origin (phyto-EPIs) reported in the running decade. We are discussing some important and successful case studies on phyto-EPIs effective against AMR phenotypes. Table 3 summarizes the list of phytochemicals, their source and effective concentrations used for inhibiting the efflux pumps of AMR bacterial strains.

**Table 3:** A summarized list of phytochemicals, their source and effective concentrations for inhibiting efflux pumps from antimicrobial resistant bacteria.

**Active Compound**	**Verified effective concentration/s**	**Plant**	**Antimicrobial resistant microbes**	**Targeted efflux pumps**	**References**
1′-S-1′-acetoxyeugenol acetate	3.12–25 mg L^−1^	*Alpinia galanga*	*Mycobacterium smegmatis* mc^2^ 155	EtBr EP	Roy et al., 2012
4-hydroxy-α-tetralone	125 μg mL^−1^	*Ammannia* spp.	MDR *E. coli*	YojI	Dwivedi et al., 2014b
Baicalein	16 μg mL^−1^	*Scutellaria baicalensis*	*S. aureus* SA-1199B	NorA	Chan et al., 2011
Berberine and Palmatine	250–1,000 μg mL^−1^ 500–100 μg mL^−1^	*Berberis vulgaris*	MDR *P. aeruginosa* (clinical isolates)	MexAB-OprM	Aghayan et al., 2017
Capsaicin	25 μg mL^−1^	*Capsicum* spp.	*S. aureus* SA-1199B	NorA	Kalia et al., 2012
Catechol	5 and 10 mg mL^−1^	*Acer saccharum*	*P. aerugenosa* ATCC 15692 and UCBPPPA14, *E. coli* ATCC 700928, *P. mirabilis* HI4320	EtBr EP	Maisuria et al., 2015
Catharanthine	25 mg L^−1^	*Catharanthus roseus*	*P. aeruginosa*	EtBr EP	Dwivedi et al., 2017b
Conessine	20 mg L^−1^	*Holarrhena antidysenterica*	*P. aeruginosa*	MexAB-OprM	Siriyong et al., 2017
Cumin-methanol extract	5 mg mL^−1^	*Cuminum cyminum*	*S. aureus* MRSA OM505	LmrS	Kakarla et al., 2016
Essential oil	5 μl mL^−1^	*Salvia fruticosa*	*S. epidermidis* (clinical isolates)	Tet(K) EP	Chovanova et al., 2015
Essential oil	170.6 μl mL^−1^	*Chenopodium* *Ambrosioides*	*Staphylococcus aureus IS-58*	Tet(K) EP	Limaverde et al., 2017
Gallotannin	12.1–97.5 μg mL^−1^	*Terminalia chebula*	MDR uropathogenic *E. coli*	EtBr EP	Bag and Chattopadhyay, 2014
Indirubin	1.25 and 2.5 mg L^−1^	*Wrightia tinctoria*	*S. aureus* SA-1199B	NorA	Ponnusamy et al., 2010
Kaempferolrhamnoside	1.56 mg L^−1^	*Persea lingue*	*S. aureus* SA-1199B	NorA	Holler et al., 2012
Lysergol	10 μg mL^−1^	*Ipomoea muricata*	*E. coli* MTCC1652 and KG4	YojI	Maurya et al., 2013
Olympicin A	50 μM	*Hypericum olympicum*	*S. aureus* 1199B	NorA	Shiu et al., 2013
Sarothrin	100 μM	*Alkanna orientalis*	*Staphylococcus aureus* NCTC 8325-4	NorA	Bame et al., 2013
Ursolic acid and derivatives	25 and 50 μg mL^−1^	*Eucalyptus tereticornis*	MDR *E. coli* (KG4)	AcrA/B, MacB, TolC and YojI	Dwivedi et al., 2014a

One of the potent EPIs, the anti-hypersensitive alkaloid reserpine was isolated from *Rauwolfia vomitoria* (Stavri et al., 2007). Similarly, EP inhibitory activity of gallotannin (1,2,6-tri-O-galloyl-b-D-glucopyranose) isolated from hydro-alcoholic extracts of *Terminalia chebula* fruits was demonstrated by Bag and Chattopadhyay (2014) against MDR uropathogenic *E. coli*. Gallotannin induced a 2- to 4-fold reduction in minimal inhibitory concentration (MIC) of test antibiotics via inhibiting the ethidium bromide (EtBr) pump (Table 3). As EtBr is a known EP-substrate, inhibition of EtBr efflux backs the postulated EP-inhibitory activity of gallotannin (Bag and Chattopadhyay, 2014). Methanolic sap extracts of *Acer saccharum* was evaluated for its drug efflux inhibitory potentials against *P. aeruginosa* (ATCC 15692 and UCBPPPA14), *E. coli* (ATCC 700928) and *P. mirabilis* (HI4320) confirmed via monitoring the EtBr efflux (Maisuria et al., 2015).

A clavine alkaloid lysergol from *Ipomoea muricata* was evaluated against AMR *E. coli* strains (MTCC1652 and KG4) to test its EP inhibitory potentials, and strong activities (higher than the standard reserpine) were exhibited by lysergol and its derivative, 17-O-3″,4″,5″-trimethoxybenzoyllysergol (Maurya et al., 2013). Authors also reported the inhibitory activities of this compound against ABC pump YojI in *E. coli* (Maurya et al., 2013). Similarly, falcarindiol, isolated from *Levisticum officinale* exhibited EPI activities against the Gram-negative strains (Garvey et al., 2011).

On the similar lines, Dwivedi et al. (2017b) reported the antibiotic-potentiating activities of catharanthine against superbug *P. aeruginosa*. The investigation involved *in silico* docking followed by the *in vitro* evaluation revealed that catharanthine potentiates the activity of tetracyclin and streptomycin, as confirmed by a reduced MIC, and acts as a potent EPI (Dwivedi et al., 2017b). A pentacyclic triterpenoid ursolic acid from leaves of *Eucalyptus tereticornis* described as a precursor of putative EPI was evaluated against MDR *E. coli* (KG4), two promising semi-synthetic, esterified derivatives of ursolic acid, 3-O-acetyl-urs-12-en-28-isopropyl ester and 3-O-acetyl-urs-12-en-28-n-butyl ester and the parent compound exhibited better EP inhibitory potencies than the standard reserpine (Dwivedi et al., 2014a). The molecular docking confirmed the targets of these compounds as AcrA/B, MacB, TolC, and YojI (Dwivedi et al., 2014a). Similarly, two alkaloids isolated from roots and rhizomes of *Berberis vulgaris*, the barberine and palmatine showed potent EP inhibitory efficacies against *P. aeruginosa* isolated from burn infections (Aghayan et al., 2017).

Phenylpropanoids from the *n*-hexane and chloroform fractions of *Alpinia galanga* exhibited EP inhibitory activities against *Mycobacterium smegmatis* mc^2^ 155ATCC 700084 (Roy et al., 2012). A dose-dependent EP inhibition was observed with 1′-S-1′-acetoxyeugenol acetate (Roy et al., 2012). Mukanganyama et al. (2012) examined another mycobacterial member *Mycobacterium aurum* A+ against a naphthoquinone diospyrine isolated from *Diospyros montana* along with its derivatives. Two derivatives proved highly potent EPI and allowed bacterial cells to accumulate high concentrations of ciprofloxacin (Mukanganyama et al., 2012).

The acylphloroglucinol isolated from *n*-hexane fractions from *Hypericum olympicum*, olympicin-A showed promising activities against *S. aureus* (Shiu et al., 2013). The radiometric accumulation assay of the strain overexpressing NorA pump indicated the enhanced accumulation of (14)C-enoxacin, thus confirming efflux inhibition (Shiu et al., 2013). Two coumarins [5,7-dihydroxy-6-(2-methylbutanoyl)-8-(3-methylbut-2-enyl)-4-phenyl-2H-chromen-2-one and 5,7-dihydroxy-8-(2-methylbutanoyl)-6-(3-methylbut-2-enyl)-4-phenyl-2H-chromen-2-one] obtained from floral buds of *Mesua ferrea* were assessed against NorA-overexpressing *S. aureus* 1199B and the clinical isolate MRSA 831 (Roy et al., 2013). Linoleic acid isolated from ethanolic extracts of *Portulaca oleracea* showed efflux inhibitory potential at 64 mg L^−1^ concentration, equivalent to reserpine when quantified against MRSA (RN4220/pUL5054: erythromycin resistant, over-expressing MsrA ABC EP, Chan et al., 2015). In search of the drug-resistance reversal agents, dos Santos et al. (2018) assessed caffeic acid and gallic acid against four strains of *S. aureus*; 1199 as a wild type strain, 1199B as NorA harboring fluroquinolone resistant, IS-58 possessing TetK pump and RN4220 possessing MrsA pump. The study confirmed caffeic acid as a potent AMR-reversal agent, as it effectively inhibited MrsA and NorA EPs from *S. aureus* strains RN4220 and 1190B, respectively (dos Santos et al., 2018). In another interesting study, Kakarla et al. (2016) reported LmrS inhibitory activities of *Cuminum cyminum*. The study revealed that the cumin inhibits the LmrS mediated transport of drugs resulting in growth inhibition of MRSA clinical isolate in a dose-dependent manner (Kakarla et al., 2016).

Traditionally, most of the investigations were aimed at identifying EPIs for Gram-positive strains for reversing their AMR characters with very few reports against Gram-negative members. This can be because Gram-negative bacteria are more difficult targets then their positive counterparts due mainly to the presence of powerful EPs and other effective membrane barriers (lipophilic layer) averting them from external impacts (Stavri et al., 2007; Prasch and Bucar, 2015). Though, some approaches have emerged in recent years for improving antibiotic-penetration across the permeability membranes of Gram-negative bacteria such as the inhibition of new accessible target, identification of uptake pathways and the “Trojan Horse” approach (achieving fast or facilitated antibiotics uptake), establishing the rules of permeation (for predicting whether elevated uptake or reduced efflux would be the most efficient way for increasing the potency of specific antimicrobial class) and identifying potent EPIs, last one being probably the most potent (Zgurskaya et al., 2015).

Recently, Bruns et al. (2017) successfully inhibited EmrD-3 pump-mediated drug efflux from a Gram-negative bacterium *Vibrio cholerae* by garlic extract and its bioactive compound, allyl sulfide. At relatively low concentrations, the extract seems to target the EmrD-3 pump, but at higher garlic extract concentrations, the respiratory chain was affected. This example confirms targeting the energization of the efflux system by plant compounds as a potential strategy for drug efflux inhibition (Bruns et al., 2017).

Further, the MFS conserved sequence motifs, present across the entire superfamily, provide vital information regarding alignments of MFS transporter sequences (at least motif containing region), which may help in understanding the structural templates and actual binding events achieved via these transporters. Molecular dynamic simulation (MDS) studies of VMAT2 multidrug transporter (MFS family) revealed the presence of two domains of six trans-membrane helices (Yaffe et al., 2013). The trans-membrane residues at anchoring sites are identified as hinge points, at which straightening and flexing movement of helices occur, required for transport. These anchor point residues are highly conserved throughout the MFS family (Yaffe et al., 2013) and are emerging targets for drug efflux inhibition. Recent advances in scientific and technological arena have added significant in-depth understandings of the structural and biochemical basis of drug efflux, substrate profiles, molecular regulation and inhibition of major EPs.

Active EPs play a critical role in intrinsic and elevated drug resistance acquired via overproduction or over-activation of pumps in Gram-negative bacteria, and the development of clinically useful EPIs or new antibiotics to bypass pump-effects continues to be a challenge in combating Gram-negative bacterial infections (Li et al., 2015). As practically all the antibiotics are susceptible to active drug efflux, the potent EPIs can target these pumps antagonistically and can make old antibiotics effective again (the phenomenon known as re-sensitization). Besides, considering the fact that several antimicrobial agents like lipophilic penicilines, many glycopeptides, oxazolidinones, macrolides and lipopeptide daptomycin are effective in treating only Gram-positive bacterial infections and their poor potencies against Gram-negative pathogens is at least partially due to their active drug efflux, novel and potent EPIs are needed to significantly broaden the range of these antimicrobial agents. All this clearly indicates that EPIs have tremendous potential in adjunctive therapies along with the known but otherwise ineffective antibiotics ultimately reducing the emergence of AMR and virulence (Opperman and Nguyen, 2015). But developing novel and potent EPI is difficult and needs to overcome several hurdles such as choice of antibiotics for potentiation and matching the pharmacological properties of EPI-antibiotics pair (Opperman and Nguyen, 2015; Zgurskaya et al., 2015).

Considering the serious threats posed by the Gram-negative bacteria and their drug-resistance nature, more investigations aiming to target them with the novel, alternative and effective approaches including exploration of natural products are coming up. Though there are limited success stories, but they may lay the foundation for developing potent EPIs to avert the AMR phenotypes with the help of natural sources.

## *In silico* Molecular Dynamics Simulations (MDS) to Screen and Develop EPIs

The MDS is a commanding approach for computational validation and to support the hypothesized mechanisms of EPs and EPIs (Nikaido and Takatsuka, 2009). It has made possible to simulate the membrane protein complex structures with micro-second time-scales. The MDS approach along with molecular docking and other *in silico* tools are successfully utilized for screening and prediction of molecular interactions between potential EPs and their corresponding inhibitors (Jamshidi et al., 2016). This has led to the unraveling of the mechanism how drug efflux systems recognize and transfer specific molecules; thus helping researchers in challenging the efflux-mediated resistance and finding appropriate EPIs for improvement in antibiotics efficacy during AMR (MDR/XDR) infections (Collu et al., 2012; Nakashima et al., 2013). There are several recent reports describing the successful applications of these *in silico* approaches for screening and identifying potent EPIs of plant origin (Bhaskar et al., 2016; Jamshidi et al., 2016; Mangiaterra et al., 2017; Verma et al., 2017).

**Figure 4:**
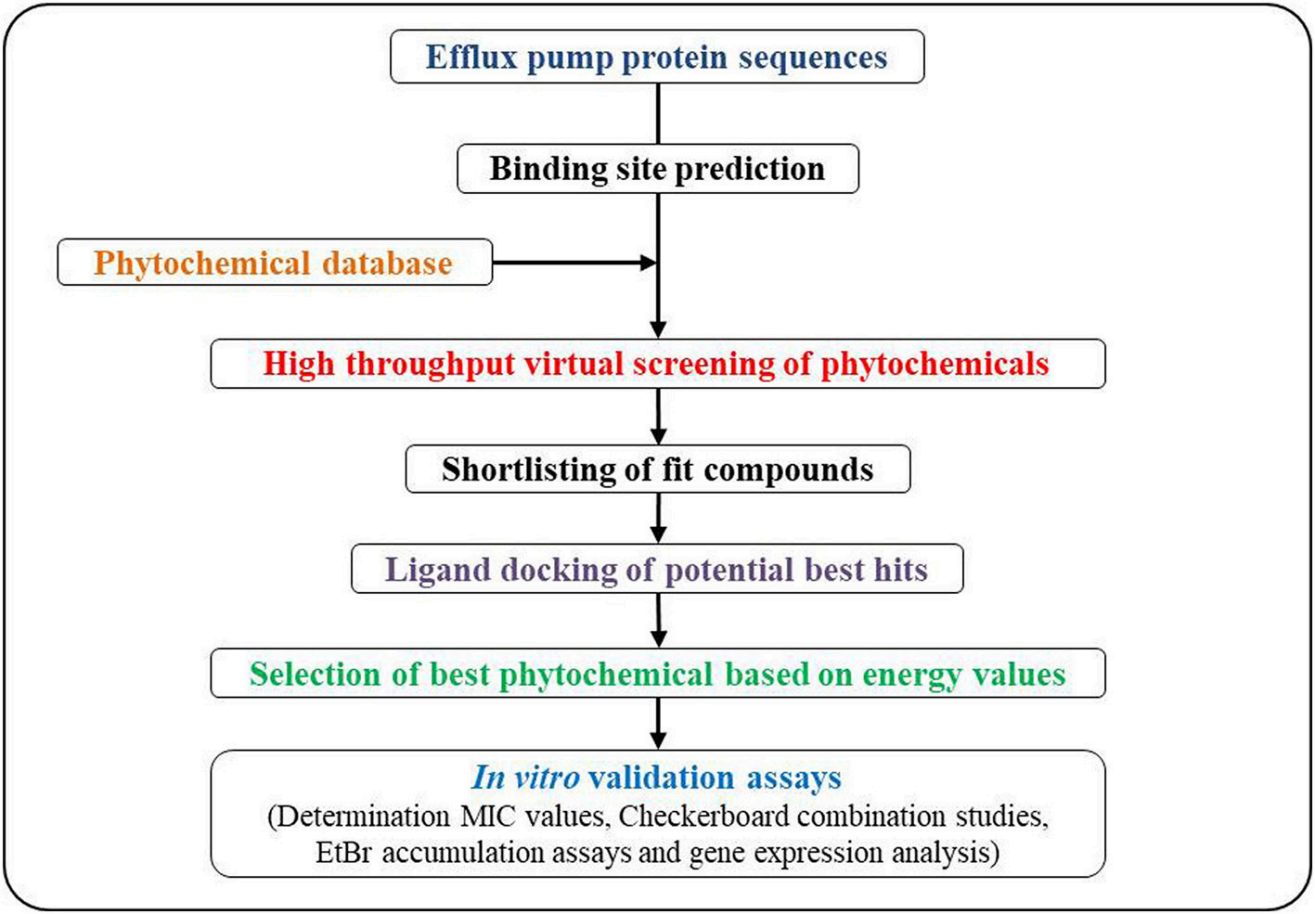
A general scheme for *in silico* molecular dynamics simulations approach for screening and developing plant based efflux pump inhibitors.

A general scheme for MDS-based approaches is depicted in Figure 4. Briefly, it starts with the identification of three-dimensional structures of potential EP-binding sites (pockets). The next step is the prediction of trans-membrane segments from protein sequences. The predicted structure can then be checked for its stereochemical properties by analyzing the overall and residue-by-residue geometry. The modeled protein structure is then reduced with the solvent implied by chimera programs (http://www.cgl.ucsf.edu/chimera/, Pettersen et al., 2004) and the projected protein structure can be used for its interaction with potential EPI molecules. The three-dimensional structure of the EPI is then explored to attain perfect and stable EPI-EP complex. Potent tools for docking studies include AutoDock (http://autodock.scripps.edu/, Morris et al., 2009), and SwissDock (http://www.swissdock.ch/, Grosdidier et al., 2011). Such automated docking tools can predict the exact binding position of the candidate drug-molecule to the receptors, and provides vital information about exact amino residues taking part in bond-formation with potential drug(s), their bond lengths and type and other interactions adding to the stability of the docking complexes.

Recently, Kesherwani et al. (2017) used high throughput virtual screening of natural compounds against NorM, a MATE transporter from *N. gonorrhea* followed by flexible docking. Authors performed molecular simulation in a membrane environment for investigating the stability and binding energy of top lead compounds, and identified a phytomolecule from *Terminalia chebula* with higher binding free energy than the substrates (rhodamine 6 g, ethidium). The compound successfully blocked the disruption of Na^+^-coordination along with an equilibrium state bias toward occlude state of NorM transporter, ultimately blocking the extrusion of antimicrobial drugs via inhibiting the NorM transporter in drug-resistant *N. gonorrhea*.

Similarly, Suriyanarayanan and Sarojini (2015) analyzed EPI potentials of plant-derived flavonoid quercetin in bringing down the drug efflux via inhibiting the EmrE, a transporter belonging to SMR family from *E. coli*. Authors used *in silico* approaches and molecule docking approaches. The docking analysis of quercetin with EP-protein showed the importance of residues for function and stability, and notably quercetin showed best interactions as compared to the compounds like verapamil, reserpine, chlorpromazine, and carbonyl cyanide m-chloro phenylhydrazone. MDS confirmed the stability of quercetin-Mmr complex, which insights the potential of quercetin as a non-antibiotics adjuvant for treatment of bacterial infection via reducing the drug efflux from bacteria.

Mangiaterra et al. (2017) identified two phyto-EPIs using *in silico* high-throughput virtual screening. Molecular docking revealed these two compounds morelloflavone and pregnan-20-one derivative as inhibitors of MexAB-OprM EPs of *P. aeruginosa* and supportive *in vitro* assays confirmed their synergism with ciprofloxacin (Mangiaterra et al., 2017).

Molecular docking plays a crucial role and help in defining drug-protein interactions which determines whether compounds act as substrates for EP proteins. Therefore, inhibitors or modulators of EPs are well-recognized along with their comparative binding efficiencies via detailed docking analyses (Collu et al., 2012). Putative EPI activities of quercetin, plumbagin, nordihydroguaretic acid, shikonion and mangiferin were confirmed (Ohene-Agyei et al., 2014). Similarly, docking of reserpine, salvin, totarol, ferruginol along with known antibiotics to NorA revealed that all the tested compounds showed binding at large hydrophobic cleft, suggesting the substantial interactions with key-residues (Bhaskar et al., 2016). Notably, all these investigations were backed by the bioassays confirming the validity of information generated via *in silico* screening.

Owing to the importance of an instantaneous requirement of curing the XDR/MDR strains with utmost specificity, a greater understanding of exact drug-identification and its transport by MDR-EPs is important. The *in silico* MDS approach along with virtual docking and wet laboratory validation therefore can be considered as an imperative path in identifying potent phyto-EPIs.

## Molecular Interactions Underlying Inhibition of Efflux Pumps by Phyto-Therapeutics

The inhibition of active drug efflux by EPIs results into the elevated intracellular antimicrobial concentrations, and lowered or complete reversal of efflux-mediated bacterial drug resistance, prevention of microbial invasiveness by inhibiting the export of virulence-factors and shortened adaptation-time required for bacteria, prohibiting the emergence of mutant strains with high AMR (Bhardwaj and Mohanty, 2012; Sun et al., 2014). Major strategies developed for drug efflux inactivation are, first, alterations in regulatory mechanisms for activation/repression of EP gene expressions (Purssell and Poole, 2013), second, deprivation of motive forces required for working of pumps by diminishing the proton gradient (Viveiros et al., 2005; Martins et al., 2008), third structural modifications in existing antimicrobials to bypass the chemophore recognition by the EPs (Chollet et al., 2004; Rice et al., 2005), fourth disrupting the pump-functionality by averting assembly of pumps by targeting protein interfaces (Tikhonova et al., 2011); interaction between protein motifs (Hobbs et al., 2012); obstructing the exit duct (Zeng et al., 2010), and fifth, the trapping of EPs in the inactivated form by competitive binding of EPIs and cytoplasmic membrane proteins (Nakashima et al., 2013; Opperman et al., 2014; Nguyen et al., 2015; Opperman and Nguyen, 2015; Figure 3). In addition, targeting the molecular hinge structures by the conserved sequence motifs is also an emerging strategy for EP inhibition (Abdali et al., 2017). The conserved sequence motifs (7–13 residues) are characteristics of MFS family and these motifs on c-terminal end of trans-membrane helix are rich in glycine and proline, vital for promotion of hinge formation. These conserved residues are considered as major contributors in binding and transportation of respective substrates (Luo and Parsons, 2010), and therefore targeting them holds significance for drug efflux inhibition.

To identify the phyto-EPIs, some authors tried to decipher the physiological and molecular interactions involved in EP dysfunction. Sharma et al. (2010) described piperine as an inhibitor of Rv1258c, an efflux protein transporter present on cytoplasmic membrane [encoding for tetracyclin/aminoglycoside resistance (TAP-2)-like EP] in *M. tuberculosis* H37Rv. After structural prediction of the protein, further investigation revealed the binding pocket of Rv1258c. Authors showed H-bond interaction (2.06 Å) with Arg141 residue and piperine provided stable protein-ligand interaction. The findings confirmed the role of piperine in augmenting rifampicin sensitivity in *M. tuberculosis* (Sharma et al., 2010). Capsaicin also proved a potent EPI, inhibiting NorA pump of *S. aureus* (Kalia et al., 2012). The study showed the involvement of Arg98 and Ile23 residues from active binding site in the key binding interactions. The stable interaction between capsaicin and active site at the proposed orientation allows an aliphatic chain of capsaicin, extending in a hydrophobic cleft (containing residues Pro24, Phe140, Ile244, and Phe303) permitting strong hydrophobic interactions due to a lesser distance between ligand and molecule (1.7–3.2 Å). A weak H-bond formation between OH-group (from aryl moiety of capsaicin) and Arg98 was attributed for providing extra-stability to capsaicin/NorA complex (Kalia et al., 2012). Another study by Zhang et al. (2014) described the interactions between ginsenoside 20(S)-Rh2 and NorA from *S. aureus*. The stable H-bond formation between ginsenoside 20(S)-Rh2 and Gln51/Asn340/Ser226 residues at active binding site in the central cavity of protein was attributed for the inhibition of NorA pump, thus promoting accumulation of ciprofloxacin inside the bacterial cell (Zhang et al., 2014). In the similar vein, Ohene-Agyei et al. (2014) assessed five phytochemicals (plumbagin, shikonin, quercetin, mangiferin and nordihydroguaretic acid) for their EPI potentials against AcrB protein from AcrAB-TolC drug transporter. The stable H-bond formation between T monomer of AcrB with minocycline attached to binding pocket and phytochemicals was responsible for efflux inhibition by the phytochemicals (Ohene-Agyei et al., 2014). Further, the authors also postulated that the considered natural compound act as a substrate and compete with the antibiotics for drug-resistance reversal (similar to PAβN). Therefore, these natural products act as high-affinity substrate inhibitors rather than substances for trapping the EPs in an inactive state.

## Concluding Remarks

Increasing AMR in community and nosocomial settings is a big threat to human healthcare and accounts for a large number of mortalities and morbidities globally. Bacterial EPs make up a major warhead of the drug-resistant pathogens and increase and maintain the AMR via extruding or reducing the intracellular concentrations of applied antibiotics, often in a non-specific manner. The drug EPs are also emerging as chemical tools to understand molecular mechanisms underlying drug extrusion from the bacterial cells. EPs play several important physiological and molecular roles in bacterial cell survival and stress-responses. The necessity to overcome AMR has encouraged investigators to characterize resistance-inhibiting or modulating EPIs to block the drug extrusion, restoring antibacterial susceptibility and returning existing antibiotics into the clinic. The severity of the AMR is higher in Gram-negative bacteria, owing to their superior capabilities in maintaining high drug efflux levels coupled with lower intracellular levels of toxic drugs including antibiotics. MDR/XDR strains maintain their intrinsic and acquired resistance via overproduction of pumps. The development of clinically useful EPIs to bypass pump effects continues to be a challenge. Though there are some noteworthy developments in recent past aimed at reversing the AMR phenotypes including facilitation of better drug-penetration across the outer membranes of Gram-negative bacteria, and establishing new rules of permeation, identifying new and powerful EPIs seems best approach that can be explored as drug leads or in adjunctive therapies.

Several recent developments in *in-silico* MDS approaches have enabled the researchers to computationally validate and support the hypothesized mechanisms of EPs and EPIs (Suriyanarayanan and Sarojini, 2015; Ramaswamy et al., 2017; Vargiu et al., 2018). It is now possible to simulate membrane protein complex structures with micro-second time-scales. The MDS approach along with molecular docking and other *in silico* tools are successfully utilized for screening and prediction of the molecular interactions between potential EPs and their corresponding EPIs, ultimately helping in identification of the potent EPIs particularly from plant origin. However, this field is yet to be explored fully.

Considering the fact that practically all the antibiotics are susceptible to active drug-efflux, use of the potent EPIs to target and block these pumps can help in potentiating the old antibiotics effective again against a range of drug-resistant bacteria. EPIs are being looked as promising adjunctive therapies with the known antibiotics to improve their antibacterial potency at low concentrations, reduce the emergence of AMR and virulence. But developing novel and potent EPI is not easy and needs to overcome several hurdles such as choice of antibiotics for potentiation and matching the pharmacological properties of EPI-antibiotic(s) pair. More comprehensive and deeper investigations are therefore needed that involve the exploring the high-throughput screening assisted by *in silico* tools for identifying the potent EPI phytomolecules and their corresponding targets. Newer studies are being undertaken for identifying phytomolecules effective in inhibiting bacterial efflux pumps via potentiation of antibiotics against pathogenic bacteria including Gram-negative pathogens. This may pave the way for identification of phyto-EPIs that can head toward clinical phases and ultimately clinical practices with an aim to contain the AMR.

## Author Contributions

VK conceived the idea. VS, TK, RB, RS, and VK wrote the manuscript. All the authors made substantial contribution to the work and approved it for publication.

### Conflict of Interest Statement

The authors declare that the research was conducted in the absence of any commercial or financial relationships that could be construed as a potential conflict of interest.
